# Weights and Measures

**Published:** 1879-09

**Authors:** 


### WEIGHTS AND MEASURES.

Wheat flour, one pound is one quart.
Indian meal, one pound two ounces are one quart.
Butter, when soft, one pound is one quart.
Loaf sugar, broken, one pound is one quart.

White sugar, powdered, one pound one ounce are one quart.

Best brown sugar, one pound two ounces are one qt.

Ten eggs are one pound.

Flour, eight quarts are one peck.

Flour, four pecks are one bushel.

Sixty grains make one drachm, eight drachms make one ounce, twelve ounces make a pound, in medicine.

